# Primary triage nurses do not divert patients away from the emergency department at times of high in-hospital bed occupancy - a retrospective cohort study

**DOI:** 10.1186/s12873-016-0102-5

**Published:** 2016-09-22

**Authors:** Mathias C Blom, Karin Erwander, Lars Gustafsson, Mona Landin-Olsson, Fredrik Jonsson, Kjell Ivarsson

**Affiliations:** 1 IKVL/Avd för medicin, Universitetssjukhuset, Hs 32, EA-blocket, plan 2, 221 85 Lund, Sweden; 2 Helsingborgs lasarett, IK-enheten, S Vallgatan 5, 251 87 Helsingborg, Sweden; 3 Pre- och intrahospital akutsjukvård, Helsingborgs lasarett, S Vallgatan 5, 251 87 Helsingborg, Sweden

**Keywords:** Emergency medicine, Bed occupancy, Emergency Department revisits, Triage

## Abstract

**Background:**

Emergency department (ED) overcrowding is frequently described in terms of input- throughput and output. In order to reduce ED input, a concept called primary triage has been introduced in several Swedish EDs. In short, primary triage means that a nurse separately evaluates patients who present in the Emergency Department (ED) and either refers them to primary care or discharges them home, if their complaints are perceived as being of low acuity. The aim of the present study is to elucidate whether high levels of in-hospital bed occupancy are associated with decreased permeability in primary triage. The appropriateness of discharges from primary triage is assessed by 72-h revisits to the ED.

**Methods:**

The study is a retrospective cohort study on administrative data from the ED at a 420-bed hospital in southern Sweden from 2011–2012. In addition to crude comparisons of proportions experiencing each outcome across strata of in-hospital bed occupancy, multivariate models are constructed in order to adjust for age, sex and other factors.

**Results:**

A total of 37,129 visits to primary triage were included in the study. 53.4 % of these were admitted to the ED. Among the cases referred to another level of care, 8.8 % made an unplanned revisit to the ED within 72 h. The permeability of primary triage was not decreased at higher levels of in-hospital bed occupancy. Rather, the permeability was slightly higher at occupancy of 100–105 % compared to <95 % (OR 1.09 95 % CI 1.02–1.16). No significant association between in-hospital bed occupancy and the probability of 72-h revisits was observed.

**Conclusions:**

The absence of a decreased permeability of primary triage at times of high in-hospital bed occupancy is reassuring, as the opposite would have implied that patients might be denied entry not only to the hospital, but also to the ED, when in-hospital beds are scarce.

**Electronic supplementary material:**

The online version of this article (doi:10.1186/s12873-016-0102-5) contains supplementary material, which is available to authorized users.

## Background

Emergency Department (ED) overcrowding has received considerable attention in the literature [1–3]. ED overcrowding is defined as a situation where the need for emergency services exceeds available resources, and its causes have been divided into input, throughput and output factors [4], of which the last have been suggested to be the most influential [1, 5]. Our group recently showed that scarcity of in-hospital beds (i.e., hospital crowding) not only increases ED length of stay (EDLOS) [6], but also causes more patients to be discharged from the ED rather than being admitted to the hospital [7, 8].

Several strategies aimed at reducing ED overcrowding through managing ED input- and throughput factors have been proposed [9]. These include fast-track service lines [9, 10], adding a physician to triage [10–13], test ordering by nurses [9, 10, 14, 15] and introducing primary care professionals in hospital EDs [16]. Other strategies aim at improving discharge planning and follow-up for patients with chronic diseases [17–19], and still others have been directed at diverting patients away from the ED [20]. In order to decrease the inflow of non-urgent patients into the ED, such a strategy has been implemented in the county council of Region Skåne in southern Sweden. The concept is called “primary triage” and its essence is that a nurse evaluates patients who are considered non-urgent upon registration in the ED. After the assessment, the nurse could admit patients to the ED, refer them to primary care or discharge them home (often with medical advice).

## Methods

### Aim

The aim of the present study is to evaluate whether the permeability of primary triage decreases at times of high in-hospital bed occupancy (i.e., whether patients are increasingly denied entry into the ED, by primary triage). An association between in-hospital bed occupancy and decreased permeability in primary triage would be worrisome, as that could suggest that nurses in primary triage deny patients evaluation by an ED physician when knowing that hospital beds are scarce. A secondary aim is to evaluate the appropriateness of discharges from primary triage by investigating whether the proportion of patients making an unplanned 72-h revisit to the ED is associated with the level of in-hospital bed occupancy.

### Study design

The study was conducted as a retrospective cohort study on administrative data from the ED at a 420-bed hospital in southern Sweden.

### Inclusion criteria

All patients registered in the ED information system Patientliggaren® in 2011–2012 and who were assessed in primary triage were included in the study.

### Sample size calculation

In order to limit bias, the study material was not subject to further restrictions. Post-hoc power calculations were performed to determine the number of strata (see cut-offs in the “variables” section) of in-hospital bed occupancy to use for group comparisons (α = 0.05, 1-β = 0.80) [21]. Absolute differences of 5 % in the proportion of patients admitted to the ED and 2 % in the proportion of patients revisiting were considered clinically meaningful for study purposes. The magnitude of the differences was arrived at by a consensus decision in the study collaboration. Sample sizes allowing for 10 events per predictor were considered appropriate for multivariate analysis [22].

### Setting

Helsingborg general Hospital is one of four hospitals providing 24/7 emergency care in Region Skåne in southern Sweden. Its ED serves a population of around 250,000, which expands to more than 300,000 in the summer due to tourism. It is an academic teaching hospital, providing education for medical students and Emergency Medicine residents. The annual ED census is around 60,000, with approximately 15 % of patients arriving by ambulance.

Upon arrival to the ED, patients are registered in the information system Patientliggaren®. Until 1^st^ January 2012, registration was performed by a nurse in the “spot-check” facility. The nurse did not measure vital parameters or conduct any physical examination, beyond recording the main complaint and a short anamnesis. The spot-check nurse could refer patients either directly to the ED, or (if their complaint was considered benign) to primary care without further assessment in the ED. If unsure whether the patient should be assessed in primary care or in the ED, the nurse could refer patients to primary triage, situated in the same physical facilities as the ED. Primary triage was staffed by a nurse who was able to conduct physical examinations and order laboratory tests. Beginning January 1, 2012, the task of registration was delegated to a secretary and the spot-check facility ceased to be. The secretary could not refer patients to primary care, but was limited to admitting patients directly to the ED or referring them to primary triage. Strict guidelines were developed for the secretary to follow (Table 1). After evaluating patients, the nurse in primary triage could admit them to the ED, refer them to primary care or discharge them home. To aid her decision, the decision-support “Triagehandboken” [23] was available in print and electronically. Nurses in primary triage could consult one of the ED physicians when in doubt, but no physician was on permanent duty in primary triage. Primary triage nurses could be asked to assist staff inside the ED during the entire study-period. Primary triage could also be bypassed at times it was experiencing long queues. Patients who were referred to the ED by a physician were directly admitted to the ED after registration and hence bypassed primary triage. Patients arriving by ambulance were admitted to the ED directly (see Additional file 1 for a schematic picture of the ED front-end organization). Patients who were referred to primary care from spot-check or from primary triage were guaranteed a medical evaluation by a nurse in primary care the same day or the day after (depending on hours of primary care availability, generally until 5 pm). One primary-care facility would accept patients outside office hours (until 8 pm), but was located 15 min away by car. Hence patients often resented primary triage nurses’ advice to contact this facility.

**Table 1:** Criteria applied to direct patients to primary triage (used by secretary)

All the criteria below need to be fulfilled before a patient can be referred to primary triage
Age >1 and < 70
Fully awake, without dyspnoea, pallor or sweatiness
Self-ambulating without problems
5 or fewer patients waiting for primary triage
Each of the following groups of patients is directly admitted to the ED after registration
Dyspnoea
Chest pain
Abdominal pain
Patients with known cancer
Foreign body
Known atrial fibrillation (where the patient suspects relapse)
Chronic bowel disease
Problems related to nasogastric tubes, catheters and plasters
Scrotal pain
Urinary obstruction or haematuria
Revisits (planned and unplanned)

After being admitted to the ED, patients underwent secondary triage (an algorithm for prioritizing patients depending on vital parameters and main complaints, similar to what is used in most EDs worldwide). During the study period, the 4-level triage system “medical emergency triage and treatment system” (METTS) was used in secondary triage [24, 25]. From secondary triage, patients were directed to separate units for Surgery, Orthopaedics, Medicine, Otolaryngology, gynaecology, paediatrics, ophthalmology and psychiatry in a triage-to-specialty model. A complementary unit staffed by emergency physicians capable of handling various complaints, except for psychiatric, otolaryngologic, ophthalmologic and paediatric (medicine) complaints, was introduced in 2010 and operates from 8 am to 11 pm daily.

### Data sources

Data on in-hospital bed occupancy was retrieved from an occupancy database used by hospital management for quality assurance activities. Occupancy was measured as the number of occupied beds divided by the number of available beds (i.e., staffed beds) in the hospital. The data source is the hospital administrative system used for billing (PASiS). The database is updated at the beginning of every hour by an application developed by the hospital informatics unit (QlikView® software). Data on ED visits was retrieved from the ED information system Patientliggaren®. Data gathering and linking was performed by the hospital informatics unit using QlikView® software. No system crashes were reported during the study period.

### Statistics

Post hoc power calculations revealed that the study sample was large enough to detect the pre-specified differences for strata of in-hospital bed occupancy of <95 %, 95–100 %, 100–105 % and >105 % for ED admissions and <95 %, 95–100 % and >100 % for 72-h revisits. Strata were proposed prior to analysis. Since 95 % reflects the median occupancy at the hospital, <95 % was used as a commonsense reference [26]. Proportions of patients experiencing each outcome were compared across strata using Fisher’s exact test.

Binary logistic regression models were constructed in order to adjust for the effects of other factors (please see below) that may influence the outcome (admission from primary triage to the ED). Also, a sensitivity analysis was performed, using occupancy as measured 3 h prior to patient presentation (rather than at presentation) in the ED. This time interval was proposed prior to analysis and reflects the median EDLOS at the study site. Variables included in the models were: sex, age group (0–1 year, 1–18 years, 18–40 years, 40–70 years and ≥70 years), shift (0 am-8 am, 8 am-4 pm, 4 pm-0 am), time of week (Mon, Tue-Fri, Sat-Sun), registration by a nurse (rather than a secretary) upon arrival, presentation on a shift with many visits (high inflow) to primary triage and presentation on a shift with high inflow to the ED. The decision on age intervals was based on the fact that patients <1 year and ≥70 years were referred directly into the ED without passing primary triage, according to the guidelines to be followed by the secretary who replaced the “spot-check” nurse in January 2012. The time intervals used for shift reflect staffing patterns at the study site. The intervals used for time of week reflect the lower staffing during weekends and the higher patient flow on Mondays. The same occupancy levels as in the crude analysis were used in the multivariate models. Presentation on a shift with high inflow was constructed as a dichotomous variable, indicating presentation on one of the 25 % of shifts subject to most visits (adjusted for shift type). In-hospital bed-occupancy and age were considered for inclusion in the models as continuous variables, but both violated the assumption of linearity in the logit and were therefore included as the ordinal variables described above [27]. Multicollinearity testing was performed using tolerance and VIF statistics. Independent variables were manually added to the models, rather than stepwise, in order not to exclude clinically relevant variables [28]. Model fit was evaluated through Nagelkerke’s R^2^. The association between each predictor and the outcome was addressed by the -2LL and the Wald statistics. Models were screened for influential cases by addressing standardized residuals. The relatively large number of comparisons warranted application of the Bonferroni correction, yielding a level of significance of *p* = 0.006. Statistical analyses were performed in IBM® SPSS® Statistics 22. Data was anonymized before analysis.

## Results

160,462 visits were registered in Patientliggaren® 2011–2012. 37,129 visits were evaluated in primary triage and 19,829 (53.4 %) of these were admitted to the ED. Of the 17,300 cases discharged from primary triage, 1,529 (8.8 %) made an unplanned revisit to the ED within 72 h.

### Crude analysis

The proportion of visits to primary triage resulting in admission to the ED was 52.3 % at in-hospital bed-occupancy <95 %, 53.5 % at 95–100 %, 56.0 % at 100–105 % and 57.3 % at occupancy >105 % (*p* < 0.001). Post hoc power analysis indicated that the study did not have sufficient power to establish the difference between occupancy 95–100 % and the reference category. Using the occupancy as measured 3 h prior to patient presentation yielded the following proportions: 52.6 % admitted to the ED at occupancy <95 %, 53.7 % at 95–100 %, 54.8 % at 100–105 % and 55.9 % at >105 % (*p* = 0.003). Post hoc power analysis indicated that the study did not have sufficient power to establish the difference between either occupancy 95–100 % or >105 % and the reference category.

Among the 17,300 cases who were discharged from primary triage, the proportion of unplanned revisits to the ED within 72 h was 8.8 % at occupancy <95 %, 9.0 % at 95–100 % and 8.7 % at >100 % (*p* = 0.885). Using the occupancy as measured 3 h prior to patient presentation yielded proportions of 9.4 % at occupancy <95 %, 8.2 % at 95–100 % and 8.2 % at >100 % (*p* = 0.020). Post hoc power calculations indicated that the study did not have sufficient power to establish these differences. Basic descriptive statistics across each of the outcomes are shown in Table 2.

**Table 2:** Descriptive statistics across outcomes

Variable		ED admission		72 h revisits	
		No	Yes	No	Yes
Sex	Female	8232 (45.8 %)	9745 (54.2 %)	7541 (91.6 %)	691 (8.4 %)
	Male	9068 (47.3 %)	10084 (52.7 %)	8230 (90.8 %)	838 (9.2 %)
Age [Years]	0–1	82 (46.3 %)	95 (53.7 %)	79 (96 %)	3 (4 %)
	1–18	3028 (46.1 %)	3545 (53.9 %)	2797 (92.4 %)	231 (7.6 %)
	18–40	8278 (52.5 %)	7478 (47.5 %)	7590 (91.7 %)	688 (8.3 %)
	40–70	5071 (42.1 %)	6972 (57.9 %)	4559 (89.9 %)	512 (10.1 %)
	>70	841 (32.6 %)	1739 (67.4 %)	746 (88.7 %)	95 (11.3 %)
Year	2011	8942 (44.8 %)	11032 (55.2 %)	8098 (90.6 %)	844 (9.4 %)
	2012	8358 (48.7 %)	8797 (51.3 %)	7673 (91.8 %)	685 (8.2 %)
Inflow >75th percentile	High inflow p-triage	5786 (45.1 %)	7037 (54.9 %)	5234 (90.5 %)	552 (9.5 %)
	High inflow ED	3935 (44.4 %)	4935 (55.6 %)	3598 (91.4 %)	337 (8.6 %)
Shift	8 am-4 pm	6216 (45.3 %)	7500 (54.7 %)	5753 (92.6 %)	463 (7.4 %)
	4 pm-0 am	8502 (49.0 %)	8859 (51.0 %)	7784 (91.6 %)	718 (8.4 %)
	0 am-8 am	2582 (42.7 %)	3470 (57.3 %)	2234 (86.5 %)	348 (13.5 %)
Time of week	Mon	2538 (47.5 %)	2810 (52.5 %)	2325 (91.6 %)	213 (8.4 %)
	Tue-Fri	8510 (46.0 %)	9972 (54.0 %)	7789 (91.5 %)	721 (8.5 %)
	Weekend	6252 (47.0 %)	7047 (53.0 %)	5657 (90.5 %)	595 (9.5 %)
Total		17300 (46.6 %)	19829 (53.4 %)	15771 (91.2 %)	1529 (8.8 %)

### Adjusted analysis

All independent variables screened for inclusion in the multivariate models were included in the preliminary primary effects models. The interaction term of in-hospital bed occupancy*high ED inflow was significantly associated with the outcome in both models addressing the proportion admitted to the ED. This warranted stratification by high ED inflow, in addition to the analysis with the interaction term omitted.

**Fig. 1:**
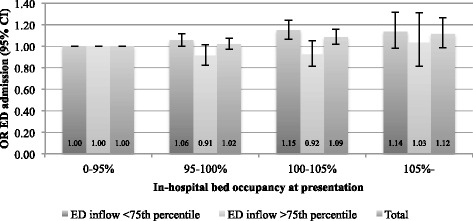
Adjusted analysis. Odds-ratio for ED admission, compared to occupancy <95 % (measured at presentation)

**Fig. 2:**
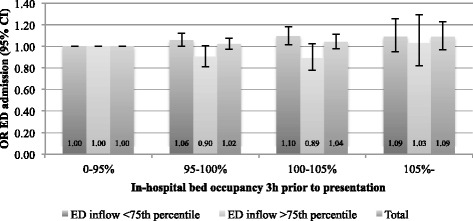
Adjusted analysis. Odds-ratio for ED admission, compared to occupancy <95 % (3 h timelag)

Neither of the analyses indicated problems with multicollinearity or multivariate outliers. The odds-ratio (OR) for ED admission for different levels of the exposure variable is shown in Figs. 1 and 2. The only significant difference in ED admission was found at occupancy 100–105 % compared to <95 % (OR 1.09 95 % CI 1.02–1.16). This effect did not remain in the sensitivity analysis. After stratifying for high ED inflow, the effect was visible in both the main analysis and the sensitivity analysis for shifts not experiencing high ED inflow, with 95 % CI for OR 1.06–1.24 and 1.01–1.18 respectively. The *p*-values from the Wald test were not statistically significant after applying the Bonferroni correction.

**Fig. 3:**
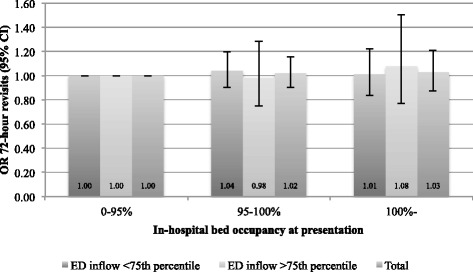
Adjusted analysis. Odds-ratio for 72-h revisit, compared to occupancy <95 % (measured at presentation)

**Fig. 4:**
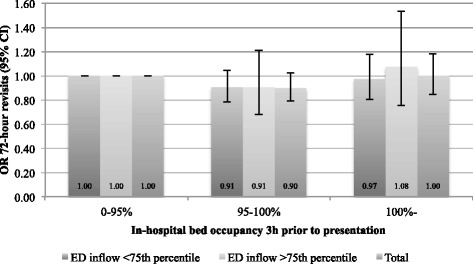
Adjusted analysis. Odds-ratio for 72-h revisit, compared to occupancy <95 % (3 h timelag)

Neither model addressing ED admission displayed any large standardised residuals. No significant differences in 72-h revisits were revealed in any of the models (see Figs. 3 and 4). The models addressing 72-h revisits displayed some disturbing residual statistics, which is why they are considered less reliable than those addressing ED admission. A detailed account of the multivariate models is given in Additional files 2 and 3.

## Discussion

Study results do not suggest that the permeability of primary triage decreases at higher levels of in-hospital bed occupancy. This holds true for occupancy measured at patient presentation as well as 3 h prior. The differences revealed in the crude analysis rather pointed towards an increased permeability of primary triage at occupancy >105 % and at 100–105 % compared to at <95 %. Even though these differences were smaller than what was considered clinically meaningful prior to conducting the study, the post hoc power analysis revealed adequate statistical power and the findings deserve some elaboration. It is possible that the results reflect a situation occurring when nurses in primary triage are asked to assist ED staff at times of high workload. The proposed causal chain is then that, when their workload is high, nurses in primary triage display a tendency to admit patients to the ED when in doubt, rather than to invest additional time in undertaking a more thorough evaluation. This would imply that the intended effect of primary triage diminishes when it is needed the most (i.e., when strain on ED staff is high). The effect of bypassing primary triage altogether could not be measured in the present study, since only patients assessed in primary triage were included.

Limitations in study power led to the collapsing of occupancy-strata for the analysis of 72-h revisits, which should be able to detect differences in the proportions revisiting the ED of 2 % and larger. The lack of a significant association between in-hospital bed occupancy and the proportion of 72-h revisits suggests that the appropriateness of discharges from primary triage was not severely affected by in-hospital bed occupancy. This would be in line with the main findings, which suggest that patients are not “bounced” by primary triage to a larger extent when in-hospital bed occupancy is high.

Since registration in Patientliggaren® is mandatory for all patients entering the facility, differential losses of data are unlikely. This is supported by the absence of system crashes during the study period. However, the generalizability of the results is impaired because of the fact that the study was conducted at a single ED. This is especially true if comparing to systems where legislation (e.g., U.S. EMTALA) prohibits diversion from the ED without proper medical screening. Even though strategies to reduce ED input by diverting patients to other levels of care are becoming less popular internationally [29], they are not uncommon in Sweden. Even though some patients presenting in the ED may do so inappropriately, the authors believe that using primary triage nurses to divert patients away from the ED may be risky, since a thorough evaluation is often required to rule out serious underlying disease. More thoroughly researched strategies to deal with less urgent patients in the ED include introducing primary care professionals [16] and fast-track services [9, 10] to the ED. Furthermore, several strategies for improving ED throughput [1, 9] and output [30–34] are available.

## Conclusions

The present study does not support the hypothesis that primary triage nurses divert more patients away from the ED at times of high in-hospital bed occupancy. This is reassuring, as the opposite would have implied that patients might be denied thorough medical assessment in the ED at times of hospital crowding. Interestingly, the permeability of primary triage appears to increase slightly at times of high demand for ED resources, which is contrary to its purpose.

## Additional files

Additional file 1: Schematic illustration of primary triage process. (PDF 31 kb) Additional file 2: Variable characteristics, multivariate models. (PDF 51 kb) Additional file 3: Variable characteristics, multivariate models, stratified by shift intensity. (PDF 66 kb)
