# Supplementary figures and images for: Primary triage nurses do not divert patients away from the emergency department at times of high in-hospital bed occupancy - a retrospective cohort study
Source: BMC Emerg Med. 2016 Sep 22;16:39. doi: 10.1186/s12873-016-0102-5 (PMC5034663; doi:10.1186/s12873-016-0102-5)

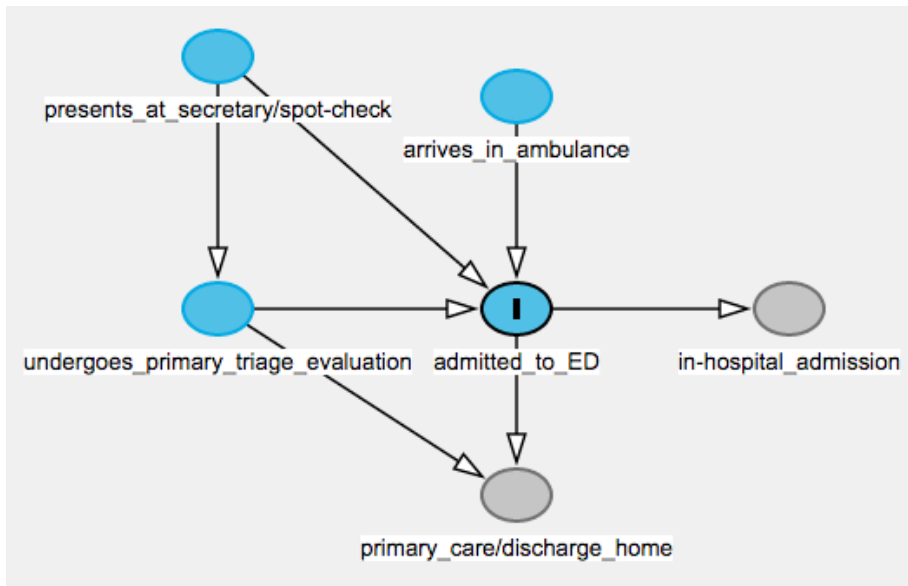

Supplement: Additional file 1: — Schematic illustration of primary triage process. (PDF 31 kb) [file 12873_2016_102_MOESM1_ESM.pdf]
