# Supplementary material for: Primary triage nurses do not divert patients away from the emergency department at times of high in-hospital bed occupancy - a retrospective cohort study
Source: BMC Emerg Med. 2016 Sep 22;16:39. doi: 10.1186/s12873-016-0102-5 (PMC5034663; doi:10.1186/s12873-016-0102-5)
Supplement: Additional file 2: — Variable characteristics, multivariate models. (PDF 51 kb) [file 12873_2016_102_MOESM2_ESM.pdf]

		In-hospital bed occ.	Regression coefficient	S.E.	Wald	Sig.	OR	95% CI for OR	
								lower	upper
ED admissions	Occ. at present. N=37,129 $R^2 = 0.026$	0-95%	Ref	Ref	Ref	Ref	Ref	Ref	Ref
		95-100%	0.021	0.025	0.722	0.395	1.021	0.973	1.072
		100-105%	0.082	0.034	6.008	0.014	1.086	1.017	1.159
		105%-	0.109	0.063	2.975	0.085	1.115	0.985	1.263
	Occ. 3h timelag N=37,118 $R^2 = 0.026$	0-95%	Ref	Ref	Ref	Ref	Ref	Ref	Ref
		95-100%	0.023	0.025	0.819	0.365	1.023	0.974	1.075
		100-105%	0.041	0.034	1.451	0.228	1.042	0.975	1.114
		105%-	0.085	0.061	1.976	0.160	1.089	0.967	1.227
72-hour revisits	Occ. at present. N=17,300 $R^2 = 0.016$	0-95%	Ref	Ref	Ref	Ref	Ref	Ref	Ref
		95-100%	0.021	0.063	0.113	0.737	1.021	0.903	1.156
		100%-	0.030	0.083	0.128	0.720	1.030	0.875	1.213
	Occ. 3h timelag N=17,294 $R^2 = 0.017$	0-95%	Ref	Ref	Ref	Ref	Ref	Ref	Ref
		95-100%	-0.106	0.065	2.641	0.104	0.899	0.791	1.022
		100%-	0.001	0.085	0.000	0.990	1.001	0.848	1.182
