# Supplementary material for: Primary triage nurses do not divert patients away from the emergency department at times of high in-hospital bed occupancy - a retrospective cohort study
Source: BMC Emerg Med. 2016 Sep 22;16:39. doi: 10.1186/s12873-016-0102-5 (PMC5034663; doi:10.1186/s12873-016-0102-5)
Supplement: Additional file 3: — Variable characteristics, multivariate models, stratified by shift intensity. (PDF 66 kb) [file 12873_2016_102_MOESM3_ESM.pdf]

			In-hospital bed occ.	Regression coefficient	S.E.	Wald	Sig.	OR	95% CI for OR	
									lower	upper
ED admission	Occ. measured at presentation (N=37,129)	Non-intense shift (N=28,259)	0-95%	Ref	Ref	Ref	Ref	Ref	Ref	Ref
			95-100%	0.054	0.028	3.664	0.056	1.056	0.999	1.117
			100-105%	0.139	0.039	12.542	0.000	1.150	1.064	1.242
		$R^2 = 0.026$	105%-	0.128	0.074	2.961	0.085	1.137	0.982	1.315
		Intense shift (N=8,870)	0-95%	Ref	Ref	Ref	Ref	Ref	Ref	Ref
			95-100%	-0.090	0.052	3.017	0.082	0.914	0.826	1.012
			100-105%	-0.079	0.066	1.422	0.233	0.924	0.813	1.052
		$R^2 = 0.025$	105%-	0.034	0.122	0.078	0.780	1.035	0.815	1.313
	Occ. measured 3h prior to presentation (N=37,118)	Non-intense shift (N=28,259)	0-95%	Ref	Ref	Ref	Ref	Ref	Ref	Ref
			95-100%	0.056	0.029	3.743	0.053	1.057	0.999	1.119
			100-105%	0.091	0.039	5.326	0.021	1.095	1.014	1.184
		$R^2 = 0.026$	105%-	0.087	0.072	1.467	0.226	1.091	0.948	1.256
		Intense shift (N=8,859)	0-95%	Ref	Ref	Ref	Ref	Ref	Ref	Ref
			95-100%	-0.101	0.055	3.387	0.066	0.904	0.811	1.007
			100-105%	-0.115	0.070	2.666	0.103	0.892	0.777	1.023
		$R^2 = 0.025$	105%-	0.032	0.117	0.073	0.787	1.032	0.821	1.297
72-hour revisits	Occ. measured at presentation (N=17,300)	Non-intense shift (N=13,365)	0-95%	Ref	Ref	Ref	Ref	Ref	Ref	Ref
			95-100%	0.039	0.072	0.303	0.582	1.040	0.904	1.197
		$R^2 = 0.014$	100%-	0.011	0.097	0.013	0.910	1.011	0.837	1.222
		Intense shift (N=3,935)	0-95%	Ref	Ref	Ref	Ref	Ref	Ref	Ref
			95-100%	-0.017	0.137	0.015	0.904	0.984	0.752	1.287
		$R^2 = 0.028$	100%-	0.074	0.170	0.191	0.662	1.077	0.772	1.504
	Occ. measured 3h prior to presentation (N=17,294)	Non-intense shift (N=13,365)	0-95%	Ref	Ref	Ref	Ref	Ref	Ref	Ref
			95-100%	-0.100	0.073	1.847	0.174	0.905	0.784	1.045
		$R^2 = 0.015$	100%-	-0.026	0.097	0.074	0.786	0.974	0.806	1.178
		Intense shift (N=3,929)	0-95%	Ref	Ref	Ref	Ref	Ref	Ref	Ref
			95-100%	-0.098	0.147	0.448	0.504	0.906	0.680	1.209
		$R^2 = 0.028$	100%-	0.073	0.181	0.161	0.688	1.075	0.754	1.534
